# The impact of weight loss after bariatric surgeries on the patient’s body image, quality of life, and self-esteem

**DOI:** 10.1007/s00423-024-03568-6

**Published:** 2025-01-04

**Authors:** Ahmed AboKhozima, Mohamed H. Zidan, Hashem Altabbaa, Aliaa Selim, Mohammed Alokl, Mohamed Mourad, Ahmed Abo Elmagd, Mohamed E. G. Elsayed, Ahmed F. Emara, Georgette M. Eskander, Samar A. Amer

**Affiliations:** 1 Alexandria University, 22 El-Guish Road, El-Shatby, Alexandria, 21526 Egypt; 2 Alexandria Main University Hospital, Alexandria University, Al Mothaf, Al Mesallah Sharq, Al Attarin, Alexandria, 5372066 Egypt; 3 El-Ekbal Hospital, 10 Hassan Amin Street, Alexandria, Egypt; 4 Alexandria Medical Research Institute, Alexandria, Egypt; 5 Borg El-Arab General Hospital, Alexandria, Egypt; 6 Department of Psychiatry and Psychotherapy III, University of Ulm, Ulm, Germany; 7 Department of Psychiatry, School of Medicine and Health Sciences, Carl Von Ossietzky University Oldenburg, Oldenburg, Germany; 8 Endocrinology, Obesity and Metabolism, University Clinic for Diabetes, University of Bern, Bern, Switzerland; 9 Department of Biochemistry, Faculty of Pharmacy, Ain Shams University, Cairo, Egypt; 10 Department of Public Health and Community Medicine, Faculty of Medicine, Zagazig University, Zagazig, Egypt; 11 The Research Papyrus Lab, Alexandria, Egypt

**Keywords:** Body Image Scale, Rosenberg Self-Esteem Scale, General Patient Satisfaction Score after Bariatric Procedures, Short Form Health Survey, Bariatric Procedures, Egypt, Obesity, Metabolic and Bariatric Surgeries

## Abstract

**Objectives:**

The objective of this web-based study is to analyze the attributes of bariatric surgery cases ensuing health implications. Additionally, the study seeks to delve into the factors influencing post-bariatric psychological evaluations and the impact of various bariatric surgeries on weight loss and psycho-social assessment scores for patients who had undergone bariatric surgeries within a specific bariatric surgery center in Egypt between January 2017 and January 2024.

**Methods:**

An analytical cross-sectional study recruited 411 adults who had undergone different bariatric procedures by the same surgical team. We collected the data using a validated self-administered questionnaire that included the Body Image Scale (BIS), the Rosenberg Self-Esteem Scale (RSES), the quality-of-life score (QOLS), and the modified General Patient Satisfaction Score after Bariatric Surgeries (GSABS).

**Results:**

The most commonly performed bariatric surgery was sleeve gastrectomy (SG), accounting for 82.7% of the procedures. The majority of the patients (78%) were female, with a mean age of 35.8. Among the participants, 32.4% reported experiencing complications, and 21.2% of those individuals were still experiencing complications at the time of assessment. The BIS had a mean score of 16.54 ± 6.27, indicating an average body image perception. The RSES yielded a mean score of 20.11 ± 4.63, indicating average self-esteem, while the GSABS had a mean score of 8.08 ± 2.39, indicating an overall average level of patient satisfaction. No statistically significant differences were found between the various types of bariatric surgeries in terms of total body weight loss percentage, excess body weight loss percentage, or the timing of the intervention. However, increased time intervals from surgeries noted a significant reduction in the BIS.

**Conclusion:**

The majority of patients who underwent SG and Roux-en-Y gastric bypass (RYGB) surgeries exhibited high GSABS scores. SG patients also had high BIS scores. However, all other interventions showed normal GSABS and BIS scores. All types of surgeries resulted in normal RSES and QOLS. Furthermore, the BIS score increases with the intervention's recentness, but it significantly decreases after the second-year post-surgery. Conversely, the older the timing of the intervention, the higher the RSES score after surgery.

**Supplementary Information:**

The online version contains supplementary material available at 10.1007/s00423-024-03568-6.

## Introduction

The prevalence of obesity continues to pose significant challenges at medical, psychological, and socioeconomic levels. According to the 2019 national health survey in Egypt, more than 19.78 million adult Egyptians are currently obese, placing immense strain on healthcare services [1]. Obesity is linked to higher mortality rates and reduced life expectancy by 5 to 20 years based on factors such as age, gender, and race [2–4]. Additionally, it is associated with an elevated risk of psychological disorder [5] and a wide range of medical conditions, including type 2 diabetes, hypertension, heart disease, stroke, and various other health issues [2, 6].

Apart from the health implications, obesity leads to weight-based discrimination in society and places a burden on the healthcare system. Chronic sleep deprivation, prolonged stress, and sedentary lifestyles further deteriorate the quality of life and raise the risk of mood disorders.

Studies have shown that a 5 to 10% Excess weight loss percentage (EWL%) can significantly reduce morbidity and mortality [7]. Bariatric surgery (BS) has gained popularity since the mid-twentieth century, prompting research into various surgical techniques, their efficacy, complication rates, and long-term outcomes [8]. Bariatric surgeries have emerged as an effective approach for morbid obesity, with EWL% often used as a measure of treatment success. However, it is important to assess the impact of these surgeries on patients' psychological well-being. Kinzl et al. have indicated that a substantial number of post-bariatric patients experience dissatisfaction with their body image due to excess skin [9], highlighting the significance of evaluating bariatric surgeries from a holistic perspective.

The psychological, social, and medical burdens of obesity are key drivers for patients seeking medical intervention. Up to 60% of obese patients experience psychiatric illnesses [10, 11], which are associated with factors such as decreased self-image [12], reduced quality of life [13], and lower self-esteem [14]. While metabolic surgeries were initially aimed at weight loss to improve comorbidities and quality of life [15, 16], their effects on body image and self-esteem are complex, with limited evidence for body image improvements and variable effects on self-esteem[17, 18]. This study aims to design a comprehensive survey to assess the psycho-social effects of bariatric surgeries, weight loss, and their impact on patients' body image, quality of life, and self-esteem.

## Patients and methods

### Study design and participants

This cross-sectional study conducted in Egypt included 411 adults who had undergone bariatric surgery at a specialized center between January 2017 and January 2024. The inclusion criteria comprised adults over 18 years old, who were one month to seven years post-surgery and had undergone a thorough multidisciplinary evaluation. All participants had received bariatric procedures from the same surgical team at the center. Data collection took place between February and April 2024, and no incentives were provided to the participants. Exclusion criteria encompassed pregnant individuals, those deemed unfit to participate by healthcare authorities, participants with specific health conditions or limitations, and incomplete survey responses.

### Sample size and sampling techniques

We used the formula n = Z^2^ P (1-P)/d^2^ to determine the sample size. In this formula, n represents the sample size, Z represents the 95% confidence level, P represents the expected prevalence, and d represents the level of precision at 80%. We collected our data using Delphi, purposive, and simple random sampling techniques. Considering a 20% response rate for our online self-administered questionnaire, we arrived at a total sample size of 386, with 322 being the minimum required.

### Data collection

The study respondents provided their consent by completing questionnaires during clinic visits or their virtual follow-up evaluation. The questionnaires were distributed to patients without scheduled follow-up appointments through mass social media contact groups or scheduled follow-up clinic visits. The questionnaire was designed with both single-answer questions and multiple-answer questions. To avoid incomplete forms, all questions were mandatory, and participants were allowed to answer once using their emails to prevent duplicate responses. Participants submitted the questionnaire after providing their consent to participate. Follow-up messages and reminders were sent to increase the response rate.

The questionnaire was distributed to 3000 participants, out of which 469 responded. After excluding 58 incomplete responses, 411 were included in the study.

### Data collection tools (preparation, validation, and structure)

The questionnaire used in the study was developed and adopted from earlier studies [9, 19–24]. It was created in English and then translated into Arabic by a bilingual panel of two healthcare professionals and one qualified medical translator. Two English-speaking translators approved the back translation for accuracy, and the original panel was consulted in case of any issues.

The questionnaire's reliability and validity were assessed through a pilot study involving 15 participants. One psychiatrist, one public health and community medicine expert, and two surgeons validated the questionnaire before assessing its readability and comprehension. We excluded those 15 pilot responses. A Cronbach's alpha of 0.82 was calculated.

The study utilized a questionnaire consisting of four main sections. *The first section* collected information on demographic and health-related factors such as participants' current age, sex, occupational status, marital status, residency, co-morbidities, participants’ subjective self-assessment of their health condition, their height, their current weight, and their pre-operative ideal weight.

*The second section* focused on the participant’s history of obesity and pre-operative bariatric surgery-related factors, the history of obesity included the age at which they began to gain weight, their belief in the cause of weight gain, the number of failed weight loss trials, and their reason for seeking surgical advice. The pre-operative bariatric surgery-related factors include the reason for the surgery, the duration since the surgery, the type of surgery, the pre-operative weight, and the pre-operative BMI.

*The third section* focused on Post Bariatric Surgery (PBS) related factors such as the presence and types of complications, Subjective self-assessment, and health condition (SSAH) score, as well as subjective postoperative changes in the patient's vital parameters of chronic disease, and the effect of surgery on the need for chronic illness medications [19]. Furthermore, the effect of weight loss on physical ability, social interactiveness, libido, and the ability to work, height, shoe size, and ring measurement, were also extracted.

Finally, *the fourth section* focused on the assessment of PBS psycho-social aspects, using multiple scales including the Body Image Scale (BIS) [21], the Rosenberg self-esteem scale (RSES) [24], the Quality-of-Life score (QOLS), and the General Patient Satisfaction After Bariatric Surgery score (GSABS).

The Body Image scale (BIS) [21] aimed to assess the impact of obesity and bariatric surgeries on body image. The BIS comprises nine questions, and each question is scored from 0 to 3 based on the patient's response. The scores range from 0 to 27, with "never" scored as 0, "sometimes" scored as 1, "rarely" scored as 2, and "at a great deal" scored as 3. The scores were categorized as "low body image" for scores between 0–9, "average body image" for scores between 10–18, and "good body image" for scores between 19–27.

The Rosenberg self-esteem scale (RSES) was used to evaluate self-esteem [24]. This ten-item scale was used to assess the self-esteem of adults and other groups, with a high Cronbach alpha (0.92) indicating exceptional internal consistency. Each question was scored from 0 to 3 based on the patient's responses, with "Strongly disagree" scored as 0, "Disagree" scored as 1, "Agree" scored as 2, and "Strongly agree" scored as 3. The total score was calculated by adding the scores of all ten questions, resulting in a range of 0–30. The scores were categorized according to previous RSES interpretation models [25–29], but following the Likert scale[30]. Scores less than 15 were considered "low self-esteem", Scores between 15 and 25 were within the normal range, and scores above 25 were above average self-esteem.

We extracted the Quality of Life Score (QOLS) from the original BAROS [20], and SF-36 [22, 31] scoring systems, to highlight the effect of bariatric surgeries on the quality of life; However, we did not use the BAROS or the SF-36 to analyze our data. The questions covered all 8 key item domains from SF-36 surveys including vitality, physical functioning, bodily pain, general health perceptions, physical role functioning, emotional role functioning, mental health/emotional well-being, and social role functioning.

The QOLS was formulated by two distinct sets of questions, covering the current health condition, and the current mental and emotional well-being. The current health condition domain was formulated by two questions each scored from 0 to 2 based on the patient's responses, with "great/significant difficulty" scored as 0, "little difficulty" scored as 1, and "No difficulty at all" scored as 2, with a total score from 0–4. The current mental and emotional well-being domain contains 6 questions each scored from 0 to 3 based on the patient's responses, with “Rarely” scored as 0, "Sometimes" scored as 1, "Most of the time" scored as 2, and "All the time" scored as 3: with a total score from 0–18. Scores from both domains were added for acquiring the total QOLS that ranged from 0–24. The scores were categorized as "low QOLS" for scores between 0–8, “average QOLS” for scores between 9–16, and "above average QOLS" for scores between 17–24.

We derived a General Patient Satisfaction After Bariatric Surgery score (GSABS) by consolidating multiple patient satisfaction scores [20, 32–34]. We adapted the questions to assess overall patient satisfaction after surgery, focusing on progress, overall experience, appearance, scar-cosmetic appearance, and weight loss rates. The score is based on four questions, each scored from 0 to 3, depending on the patient's response. The total score ranges from 0 to 12, with scores between 0–4 indicating low patient satisfaction, scores between 5–8 indicating average satisfaction, and scores between 9–12 indicating good satisfaction.

All the answers given by participants and the questions asked in the survey are available in Supplementary File 1 on the online version of this article.

### Study variables

Weight loss was evaluated by calculating the Total Body Weight Loss Percentage (TBWL%) and the Excess Body Weight Loss Percentage (EWL%).

The primary BMI of the patient was compared with the current BMI to determine the EWL%. EWL% was calculated using the formula: EWL% = ((Initial BMI—Final BMI) / (Initial BMI—Ideal or Target BMI)) *100 [35].

The Total Body Weight Loss percentage (TBWL%) also known as the percent of weight change was calculated using the formula TBWL% = ((Initial Weight—Final Weight) / Initial Weight) *100 [35].

According to the IFSO, recurrent weight gain is defined as a gain of more than 30% of the initial surgical weight loss [36].

### Statistical analysis

The data collected was analyzed using SPSS version 25. We have then compared all variables with the scoring systems used to evaluate the effect of different bariatric surgeries and weight loss on patients' overall health, self-esteem, body image, patient satisfaction, and quality of life. Furthermore, the effect of weight loss on physical ability, social instructiveness, libido, and the ability to work, and Quantitative data (such as BMI) was presented as mean ± SD if normally distributed or median (IQR) if not normally distributed. To analyze the normally distributed quantitative data, the Kruskal–Wallis test was used after testing the Kolmogorov–Smirnov test. Qualitative data, such as age groups and sex, were presented as frequency and percentage, and the association between categorical variables was tested using the chi-squared test (χ^2^), and Fisher’s exact test but the association between categorical and quantitative data, Mann-Whitny U test was used.

A Pearson’s product-moment correlation was run to assess the relationship between two continuous variables if normally distributed. If not normally distributed, we used Spearman's rank-order correlation.

### Ethical considerations

The Institutional Review Board (IRB), the Ethics Committee of the research center at Alexandria University, endorsed the research study and carried it out per the Declaration of Helsinki and its amendments (IRB: 00012098). All respondents gave their informed consent before participating in the study. The questionnaire was designed to avoid questions that might be sensitive or private, and the identities of the respondents were kept anonymous throughout the study.

## Results

### Surgical interventions, demographics, and health-related issues and their surgical effect

The study included 411 participants who underwent various bariatric procedures by a single surgical team using the same surgical technique, primarily Sleeve Gastrectomy (SG) (82.7%). Other surgeries included Single Anastomosis Sleeve-Ileal Bipartition (SASI) (7.2%), Intra Gastric Balloon (IGB) (3.9%), One-anastomosis gastric bypass (OAGB) (2.9%), and Roux-en-Y Gastric Bypass (RYGB) (1.2%).

Demographically, 78% of participants were female, with a mean age of 35.8 years; 88.6% lived in urban areas (Table 1). Most experienced weight gain beginning around 19.6 years, with 79.3% starting before 30 years of age. The primary causes identified were poor eating habits (82.7%), lack of physical activity (50.9%), and genetics (46.7%). The main reasons for seeking surgery were to improve general health (81%) and self-esteem (54%) (Table 2).

**Table 1:** Demographics, and clinical characteristics of participants and their relation to Rosenberg self-esteem scale (RSES), general patient satisfaction score after Bariatric Surgeries (GSABS), and the Quality-of-Life core (QOLS)

Demographics	(*n* = 411) *n*(%)	RSES median (IQR)	GSABS median (IQR)	QOLS median (IQR)
**Current Age:** median (IQR)	35.78 (9.09)	-	-	-
Age less than 30 years	101 (24.6)	19 (16—23)	8 (6—10)	11 (10—13)
Age 30 – 45 years	258 (62.8)	21 (17—24)	8.5 (7—10)	10 (8—12)
Age more than 45 years	52 (12.7)	19.5 (17—23)	8 (6—9)	10 (7—11)
*P-*value *		0.03	0.73	< 0.001
**Sex**				
Male	88 (21.4)	19 (16—23)	9 (7—10)	10 (8—12)
Female	323 (78.6)	20 (17—24)	8 (6—10)	11 (9—12)
* P-*value **		0.81	0.02	0.155
**Residency**				
Urban	364 (88.6)	20 (17–23.7)	8 (7—10)	11 (8.25—12)
Rural	47 (11.4)	20 (17—23)	8 (6—10)	10 (8—12)
* P-*value **		0.667	0.38	0.296
**Marital Status**				
widowed/Divorced	29 (7.1)	20 (16.5—23)	8 (6—9)	10 (8.5—12)
Single	114 (27.7)	20 (16—24)	8 (7—10)	11 (9—12.25)
In a relationship/married	268 (65.2)	20 (17—23)	8 (7—10)	10 (8—12)
* P-*value *		0.857	0.08	0.025
**Occupational status**				
Occupied	229 (55.7)	20 (17—24)	8 (7—10)	10 (8—12)
Non-Occupied	182 (44.3)	20 (17—23)	8 (6—10)	11 (9—12)
* P-*value **		0.127	0.078	0.252
**Co-morbidities**				
No	162 (39.4)	20 (17—23)	8 (7—10)	10 (9—12)
yes	249 (60.6)	20 (17—24)	8 (6—10)	11 (8—12)
* P-*value **		0.646	0.728	0.66
**Patients’ subjective self-assessment of health condition (SSAH)**				
Excellent	98 (23.8)	22 (17—25)	10 (8—11)	10 (9—12)
Very good	141 (34.3)	20 (17—23.5)	9 (8—10)	10 (8—12)
Good	129 (31.4)	19 (16—23)	7 (6—9)	11 (8.75—13)
Fair	42 (10.2)	19 (16—22.2)	5.5 (4—8)	11 (8—13)
Poor	1 (0.2)	19 (19—19)	8 (8—8)	11 (11—11)
* P-*value*	-	< 0.001	0.06	0.114
**Weight:**	Median(IQR)	r*(P*)	r*(P*)	r*(P*)
Current	88 (76—103)	−0.163(0.001)	0.231(< 0.001)	−0.011(0.828)
Ideal	57.88 (52.42–64.25)	−0.20 (0.689)	0.055(0.266)	0.016 (0.752)
**BMI (kg/m**^**2**^**):**	Median (IQR)	r*(P*)	r*(P*)	r*(P*)
Current	31.64 (27.88–36.7)	−0.173 (< 0.001)	−0.284 (< 0.001)	0.011(0.826)
Pre-operative	44.81 (40.52–49.49)	−0.107 (0.03)	−0.118 (0.016)	0.057(0.398)

*(Kruskal Wallis H test)

**(Mann–Whitney U test)

r(P) (Spearman’s Correlations)

**Table 2:** Obesity and Bariatric Surgery-related history among patients

Obesity, and bariatric surgery-related history	*n* (%)
**Age at which the patient started gaining weight.** Mean (SD)	19.59 ± 9.87
Age less than 30	326 (79.3)
Age 30 – 45	82 (20)
Age more than 45	3 (0.7)
**Excess Body Weight Loss Percentage (EWL%)** Mean ± SD	52.93 ± 21.37
Less than 50%	179 (43.6)
50% or more	232 (56.4)
**Total Body Weight Loss Percentage (TBWL%)** Mean ± SD	27.62 ± 11.36
Less than 25%	166 (40.4)
From 25—< 50%	237 (57.4)
From 50—< 75%	8 (1.9)
75% or More	0 (0)
The associated comorbidities *(*n* = 162)	
Endocrine Disorders Including Diabetes or thyroid disease	63 (38.9)
Chronic Kidney Disease	6 (3.7)
Osteoarthritis	74 (45.7)
Cardiovascular disease	18 (11.1)
Behavioral or psychiatric illness	10 (6.17)
Liver Disease	4 (2.5)
Other	61 (37.6)
The patient's subjective self-reported causes for their weight gain*	
Endocrine Disorders	64 (15.6)
Absence of Physical activity	209 (50.9)
Bad eating habits	340 (82.7)
Genetic factors	192 (46.7)
Increased age with increased sedentary life	17 (4.1)
Reasons related to pregnancy and lactation (overfeeding)	100 (24.3)
Behavioral or psychiatric disorders (including periodic depression)	102 (24.8)
Drug therapy (including corticosteroids antidepressants or insulin)	52 (12.7)
**The number of times the patient has tried to lose weight**	
Less than 5	100 (24.3)
From 5 to 10	188 (45.7)
More than 10	123 (29.9)
The reason that the patient sought surgical help*	
To improve general health	333 (81)
To improve medical condition	168 (40.9)
Psychological reasons and to improve self-esteem	222 (54)
For social reasons	80 (19.5)
**Time of which intervention was done**	
Less than 2 months ago	29 (7)
From 2 to 6 months ago	124 (30.2)
From 6 months to less than 2 years ago	200 (48.6)
From 2 to 5 years ago	52 (12.7)
More than 5 years ago	6 (1.5)

*Multiple answers were allowed

Postoperatively, the median BMI decreased from 49.49 kg/m^2^ pre-op to 40.52 kg/m^2^ (Table 1). Most surgeries occurred within 6 months to 2 years, with a mean total body weight loss (TBWL%) of 27.6%, and a mean EWL% of 52.93 ± 21.37%, with 56.4% of patients having an EWL% greater than 50 (Table 2).

In the context of surgical complications, 32.4% (*n* = 133) of the participants reported experiencing a complication, out of which 21.2% (*n* = 87) were still experiencing them and 11.2% (*n* = 46) had their complications managed. The majority experienced general weakness (36.1%), and hair loss (30.8%). Only two of the 133 patients experienced major surgical complications, such as post-operative bleeding (0.7%) and postoperative intensive care unit admission.

Changes in physical measurements included shoe size (45.3% decreased slightly), ring size (52.1% decreased significantly), and height (65.2% unchanged) (Table 3). Out of 290 cases with medical comorbidities, 45.5% reported slight improvements in their conditions, 36.6% reported absolute recovery of their medical condition after a medical consultation, and 44.1% completely weaned off medications following surgery. Only 3.1% experienced worsened medical issues post-operation (Table 3).

**Table 3:** The post-bariatric surgery (PBS) changes or complications among participants after Bariatric Surgeries (BS)

PBS changes or complications among participants	*n*(%)
**Did you experience any complications after the operation?**	
o Yes, and I am still complaining of it	87 (21.2)
o Yes, but it has been managed	46 (11.2)
o No	278 (67.6)
Complications (n = 133) *	
Bone aches	5 (3.76)
Hair loss	41 (30.82)
Cholecystitis	7 (5.26)
Periodic abdominal pain	15 (11.27)
Loss of skin turgidity	1 (0.75)
Post-prandial vomiting	21 (15.79)
Dizziness	12 (9.02)
Dysphagia	1 (0.75)
Dyspepsia	12 (9.02)
General weakness and fatigue	48 (36.09)
Nausea	18 (13.53)
Dehydration	5 (3.76)
Post-prandial abdominal pain	1 (0.75)
Onychoschizia	4 (3.01)
Cosmetic and skin problems	4 (3.01)
Hypotension	3 (2.25)
Post-operative Intensive care unit admission	1 (0.75)
Anhedonia (loss of pleasure)	1 (0.75)
Dysmenorrhea	1 (0.75)
Post-operative bleeding	1 (0.75)
**The effect of Surgery on co-morbidities and the need for medications (n = 290)**	
Worsening of the medical condition with an increased need for medications	11 (3.4)
No change was noted with the same doses taken pre-operatively	52 (17.9)
Slightly decreased dosages with improvement of health condition	99 (34.1)
Absolute weaning of medications after medical consultation	128 (44.1)
**The effect of Surgery on co-morbidities (n = 290)**	
Worsening the medical condition (co-morbidities)	9 (3.1)
No change was noted	43 (14.8)
Slight improvement of medical condition	132 (45.5)
Absolute weaning of medications after medical consultation	106 (36.6)
**Changes in the shoe size**	1.19 ± 0.72
Increased	1 (0.2)
Did not change	151 (36.7)
Slightly decreased	186 (45.3)
Significant decrease (more than two degrees)	73 (17.8)
**Changes in the ring size**	0.55 ± 0.63
Increased	2 (0.5)
Did not change	25 (6.1)
Slightly decreased	170 (41.4)
Significant decrease (more than two degrees)	214 (52.1)
**Changes in the height**	1.73 ± 0.72
Increased	33 (8)
Did not change	268 (65.2)
Slightly decreased	78 (19)
Significant decrease (more than two degrees)	32 (7.8)

*multiple answers were allowed

Out of the 290 individuals on medication, the majority (*n* = 128; 44.1%) underwent a complete weaning of their medications following a medical consultation. Of these, 34.1% (*n* = 99) showed no change in their pre-operative doses, while only 3.8% (*n* = 11) reported worsening of their medical condition and an increased need for medications (Table 3).

### Psycho-psychiatric assessment parameters: Subjective self-assessment of health condition (SSAH), Body Image Scale (BIS), The Rosenberg Self-esteem Scale (RSES), Quality of Life Scale (QOLS), and General Satisfaction after Bariatric Surgery (GSABS) scores

Participants were asked to report on their health condition subjectively (SSAH). Most reported very good (34.3%) or excellent (23.8%) health, totaling 58.1%. Meanwhile, 41.5% described their health as good (31.5%) or fair (10.2%), with 0.2% rating it as poor (Table 1). Those with excellent and good health reported higher self-esteem and satisfaction but lower quality-of-life scores.

**Fig. 1:**
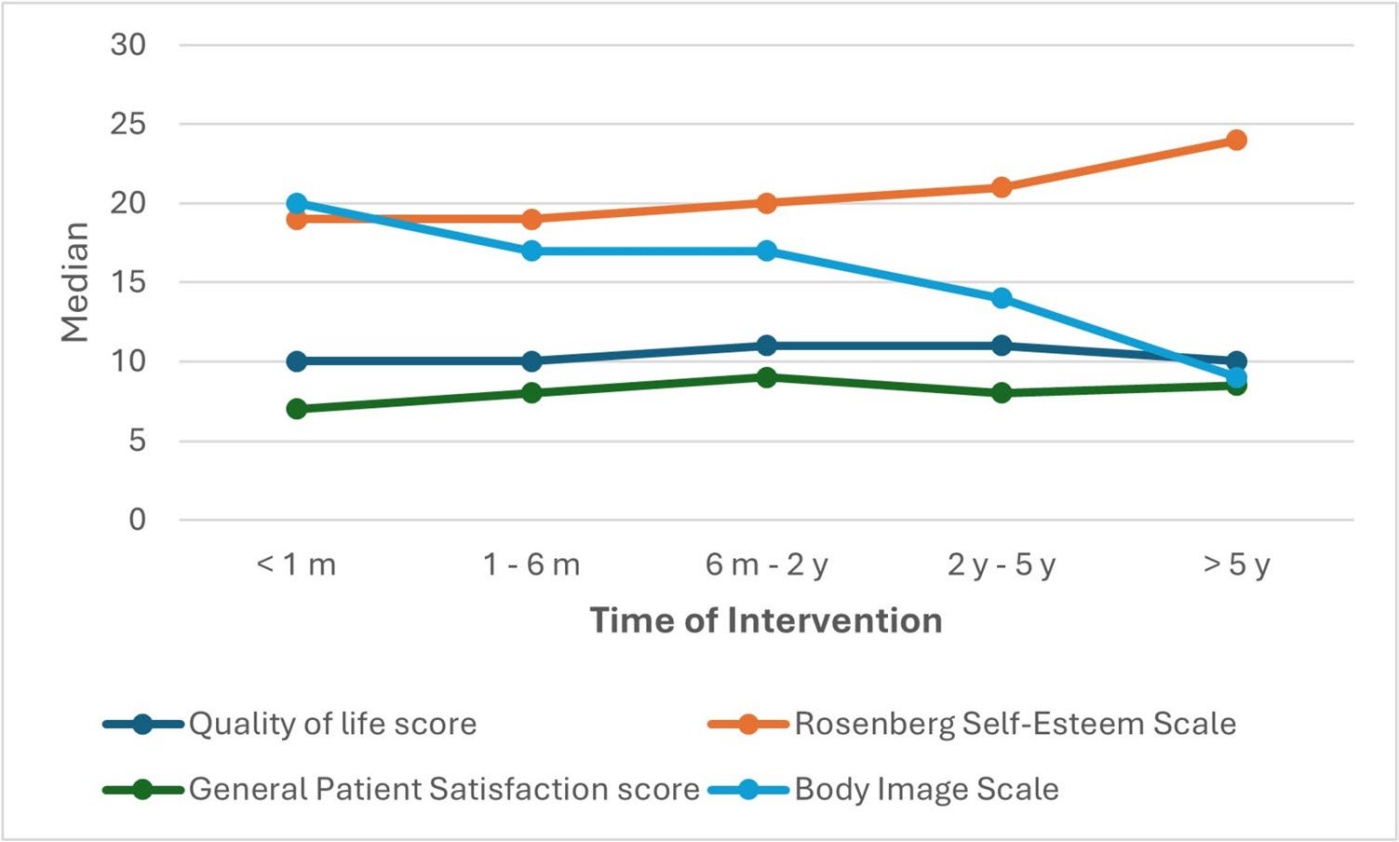
Line chart showing the correlation between the timing of intervention and the median psychological assessment scores. Note that there is no correlation between the timing interval, General patient satisfaction (GSABS), and quality of life scores (QOLS). However, as the timing of intervention increases, the median quality-of-life scores (QOLS) increase, and the median Body Image scale (BIS) decreases

Among the 411 participants, the mean Body Image Scale (BIS) score was 16.54 ± 6.27 post-bariatric surgery (Table 4), indicating average body image perception. The study found no correlation between weight loss or intervention type and BIS improvement (Table 5). However, more recent interventions were associated with higher BIS scores, with a notable decline after the second year post-surgery (Fig. 1).

**Table 4:** Table showing The post-bariatric surgery assessment scores, Body image scale (BIS), Rosenberg Self-esteem Scale (RSES), and stratification of self-esteem

Assessment Scale	**Mean** ± SD (%)
**Patient post-operative satisfaction score regarding:**	
The procedure/operation	2.58 ± 0.66
The shape of your body after the operation	2.24 ± 0.81
The appearance of your skin after the operation	1.31 ± 0.97
The rate of your total weight loss	1.95 ± 1.01
Total Score	8.08 ± 2.39
**Body Image Scale**	16.54 ± 6.27
**Rosenberg Self-Esteem Scale (RSES)**	20.11 ± 4.63
**The total classification of self-esteem**	
Low Self-esteem (0–14)	42 (10.2)
Normal Self-esteem (15–25)	313 (76.2)
High/above average self-esteem (26–30)	56 (13.6)

**Table 5:** Correlation between different aspects of weight loss, timing of intervention, type of intervention, and weight loss trials to the psycho-social assessment scores

	General Patient Satisfaction score Median (IQR)	Body Image Scale Median (IQR)	Rosenberg Self-Esteem Scale Median (IQR)	Quality of life score Median (IQR)
**Total Body Weight Loss Percentage (TBWL%)**				
Less than 25	7 (5—9)	18 (12 −22)	19 (17—23)	10 (8—12)
From 25—< 50	9 (7—10)	16 (11—21)	21 (17—24)	11 (8—12)
From 50—< 75	9 (7.5—10)	18.5 (10.75- 26)	18.5 (15.25—19.75)	10 (9—11.75)
75 or More	0	0	0	0
* P*-value*	** < 0.001**	0.146	0.228	0.498
**Excess Body Weight Loss Percentage (EWL%)**				
Less than 50%	7 (5—9)	18 (12.5—22)	19 (17—23)	10 (8—12) 11
50% and more	9 (8—10)	16 (11—20)	21 (17—24)	(8—12)
* P*-value**	** < 0.001**	**0.009**	**0.047**	0.315
**Time of which intervention was done**				
Less than 1 month ago	7 (5—9.5)	20 (12.5—23)	19 (16.5—25)	10 (8—11)
From 1 to 6 months ago	8 (6.25—10)	17 (12—22)	19 (17—22)	10 (8—12)
From 6 months to 2 years ago	9 (7—10)	17 (12—21)	20 (17—24)	11 (9—12)
From 2 to 5 years ago	8 (7 −10)	14 (10—20)	21 (17.25—24.75)	11 (9—12)
More than 5 years ago	8.5 (6.75—11)	9 (6.75—18.5)	24 (20—27.5)	10 (8.25—11)
* P*-value*	0.243	**0.043**	0.077	0.334
**Type of intervention performed**				
IGB	8 (7—10)	16.5 (13.5—20.75)	21.5 (16.25—22.75)	10.5 (9.25—12)
RYGB	9 (4.5—11)	18 (9.5—23)	20 (17.5—27)	14 (12—15)
Mini gastric Bypass	7 (4.25—10)	16.5 (10.75—21.5)	18 (16.25—20.75)	8.5 (8—12)
SASI only	7.5 (5—9.25)	15.5 (11—21.75)	20.5 (18.5—25)	10 (8—11.25)
Sleeve gastrectomy	8 (7—10)	17 (12—21)	20 (17—23)	10 (8—12)
* P*-value*	0.390	0.997	0.484	**0.049**
**Number of times the patient has tried to lose weight**				
Less than 5	8 (7—10)	16 (10—20)	19.5 (17—23)	10 (8—12)
From 5—< 10	8 (7—10)	17 (11.25—21)	20.5 (17—23)	10 (8—12)
More than 10	9 (6—10)	18 (14—23)	20 (16—24)	11 (9—13)
* P*-value*	0.905	**0.008**	0.460	0.058

*(Kruskal Wallis H test)

**(Mann-Whitny U test)

A total of 411 participants had a mean score of 20.11 ± 4.63 on the RSES (Table 4). No significant differences were found between RSES and demographic factors such as sex (*P* = 0.81), residency (*P* = 0.667), marital status (*P* = 0.857), occupational status (*P* = 0.127), or co-morbidities (*P* = 0.64) (Table 1). However, there was a significant correlation with age (*P* = 0.03), where participants aged 30–45 scored higher with a median of 21 (Table 1). Those rating their health as excellent or very good achieved higher median RSES scores of 23 and 20, respectively, compared to a score of 19 for those with fair or bad ratings.

The study also examined the relationship between RSES and weight loss metrics (TBWL% and EWL%) as well as the timing and type of intervention (Table 5). A strong negative correlation was noted between current weight and both current and preoperative BMI (*P* < 0.05), indicating that greater weight loss was associated with higher RSES scores (EWL% > 50%: *P* = 0.043). Additionally, a weak positive correlation existed between self-esteem and patient satisfaction (*P* < 0.05) (Table 6); However, there was no significance to the type of surgery to RSES (*P* = 0.484) (Table 7).

**Table 6:** The correlation between the following continuous variables

	Rosenberg Self-esteem r(*p*)	Quality of life r(*p*)	Patient Satisfaction r(*p*)	Body image scale
Current Age*	0.073 (0.139)	−0.198 (**< 0.001**)	0.013(0.793)	−0.012 (0.803)
Age at onset of obesity*	0.042 (0.397)	−0.132 (**0.008**)	0.06(0.222)	−0.085 (0.086)
Patient satisfaction*	0.175 (**< 0.001**)	0.004 (0.941)	-	−0.078 (0.116)
Quality of life*	−0.057 (0.245)	-	0.004(0.941)	0.074 (0.136)
TBWL%	−0.088 (0.075)	−0.022 (0.660)	−0.239 (**< 0.001**)	0.064 (0.195)

*(Pearson’s Correlation)

**Table 7:** Correlation between the type of intervention and different parameters including TBWL%, EWL%, General Patient Satisfaction Score After Bariatric Surgeries (GSABS), Body Image Scale (BIS), Rosenberg self-esteem (RSES), and Quality of Life Scores (QOLS). Types of interventions included Intragastric Balloon (IGB), Roux-en-Y Gastric Bypass (RYGB), One anastomosis Gastric Bypass (OAGB), and Sleeve gastrectomy (SG)

	Type of Intervention				
	IGB (*n* = 16) (%)	RYGB (*n* = 5) (%)	OAGB (*n* = 12) (%)	SASI (*n* = 30) (%)	SG (*n* = 340) (%)
**Total Body Weight Loss Percentage**					
Less than 25	10 (62.5)	2 (40)	5 (41.7)	17 (56.7)	127 (37.4)
From 25—< 50	6 (37.5)	3 (60)	6 (50)	13 (43.3)	206 (60.6)
From 50—< 75	0 (0)	0 (0)	1 (8.3)	0 (0)	7 (2.1)
75 or More	0 (0)	0 (0)	0 (0)	0 (0)	0 (0)
* P*-value* = 0.203					
**Excess Body Weight Loss Percentage**					
Less than 50%	11 (68.8)	2 (40)	4 (33.3)	17 (56.7)	140 (41.2)
50% and more	5 (31.3)	3 (60)	8 (66.7)	13 (43.3)	200 (58.8)
* P*-value* = 0.101					
**Time of which intervention was done**					
Less than 1 month ago	2 (12.5)	1 (20)	1 (8.3)	0 (0)	24 (7.1)
From 1 to 6 months ago	8 (50)	1 (20)	3 (25)	12 (40)	97 (28.5)
From 6 months to 2 years ago	6 (37.5)	2 (40)	4 (33.3)	16 (53.3)	168 (49.4)
From 2 to 5 years ago	0 (0)	1 (20)	4 (33.3)	1 (3.3)	46 (13.5)
More than 5 years ago	0 (0)	0 (0)	0 (0)	1 (3.3)	5 (1.5)
* P*-value* = 0.296					
**GSABS** *P*-value** = 0.390	8 (7—10)	9 (4.5—11)	7 (4.25—10)	7.5 (5—9.25)	8 (7—10)
**BIS** *P*-value** = 0.997	16.5 (13.5—20.75)	18 (9.5—23)	16.5 (10.75—21.5)	15.5 (11—21.75)	17 (12—21)
**RSES** *P*-value** = 0.484	21.5 (16.25—22.75)	20 (17.5—27)	18 (16.25—20.75)	20.5 (18.5—25)	20 (17—23)
**QOLS** *P*-value** = **0.049**	10.5 (9.25—12)	14 (12—15)	8.5 (8—12)	10 (8—11.25)	10 (8—12)

*(Chi-square test)

**(Kruskal Wallis H test)

Regarding Quality-of-Life Scale (QOLS) scores, no significant differences were found across most demographics, except for age and self-assessed health, where older patients reported higher scores (*P* < 0.001). Moreover, patients who reported good health conditions exhibited higher QOLS scores compared to those who reported poor health conditions (Table 1). In terms of surgical outcomes, the QOLS scores did not show statistically significant differences with weight loss metrics (TBWL% and EWL%) at the time of intervention (Table 5). Furthermore, our analysis demonstrated a weak negative correlation (*P* < 0.05) between current age (*P* < 0.001) and age at the onset of obesity *(P* = 0.008), indicating a potential influence of age on quality of life (Table 6).

General Satisfaction after Bariatric Surgery (GSABS) scores showed that Patient satisfaction averaged 8.08 ± 2.39, with higher satisfaction among males and those aged 30–45. Patients with good self-esteem scores also reported higher satisfaction (Table 1). Greater TBWL% and EWL% correlated with higher satisfaction scores, with no significant relationship found related to the timing of the intervention (Table 5). Furthermore, RYGB, SG, and IGB procedures showed higher satisfaction outcomes compared to SASI and OAGB (Table 7).

**Fig. 2:**
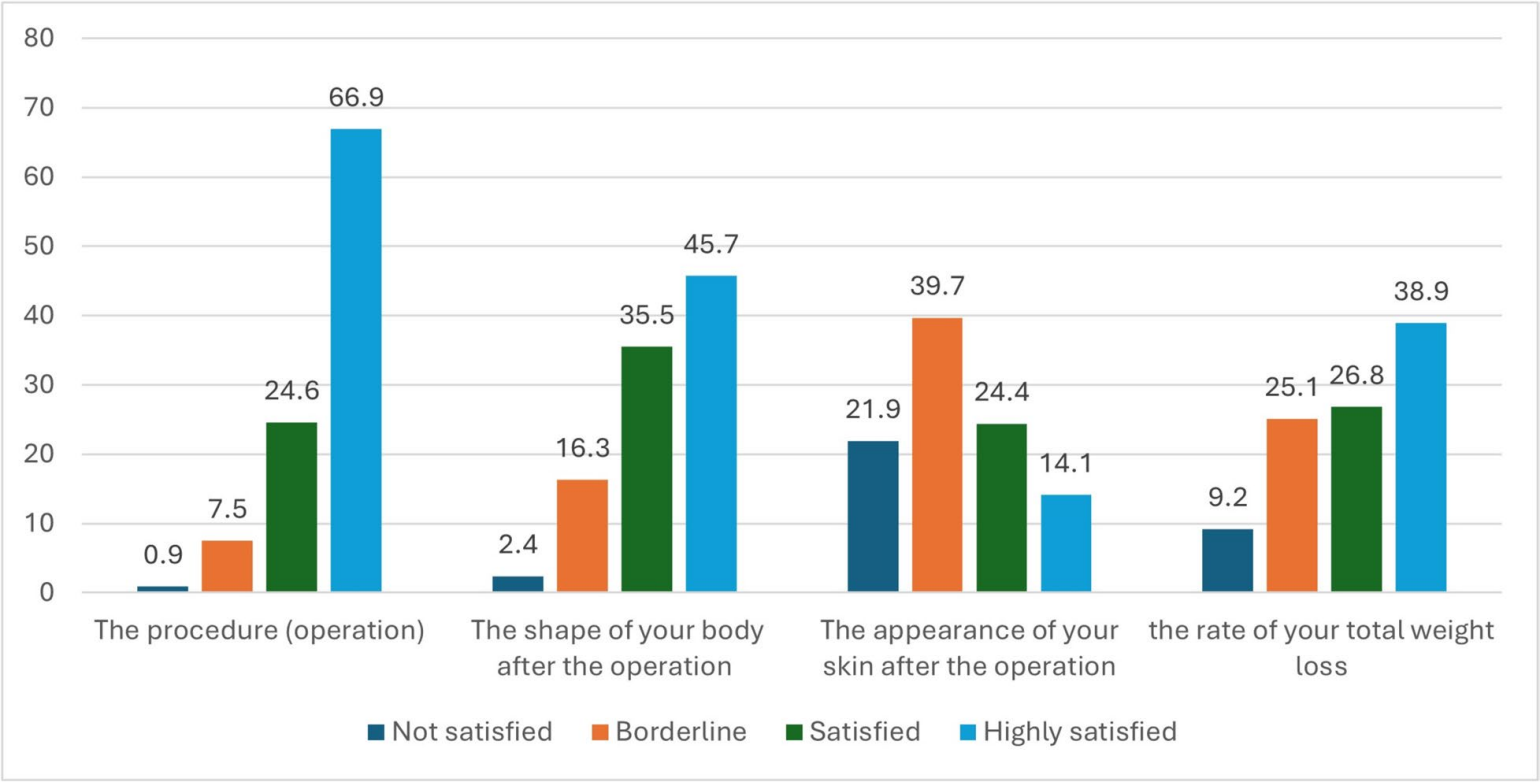
The frequency distribution of post-bariatric surgery participant satisfaction regarding the operation, body shape, Skin appearance of the skin, and total weight loss

Participants were directly asked to report their satisfaction following bariatric surgery. The results showed that 91.5% of participants were satisfied with the procedures themselves, while 81.2% were pleased with the shape of their bodies after the operation. However, only 65.7% expressed satisfaction with the overall weight loss achieved. Additionally, regarding skin appearance, 39.7% of participants felt their satisfaction was borderline, and 21.9% reported dissatisfaction (Fig. 2).

### The relationship between various bariatric surgeries, weight loss, and psycho-psychiatric assessment

There was no statistically significant difference between the types of bariatric surgeries in terms of the TBWL% and EWL or the time of intervention. However, the study noted that the more recent the intervention, the higher the BIS score.

**Fig. 3:**
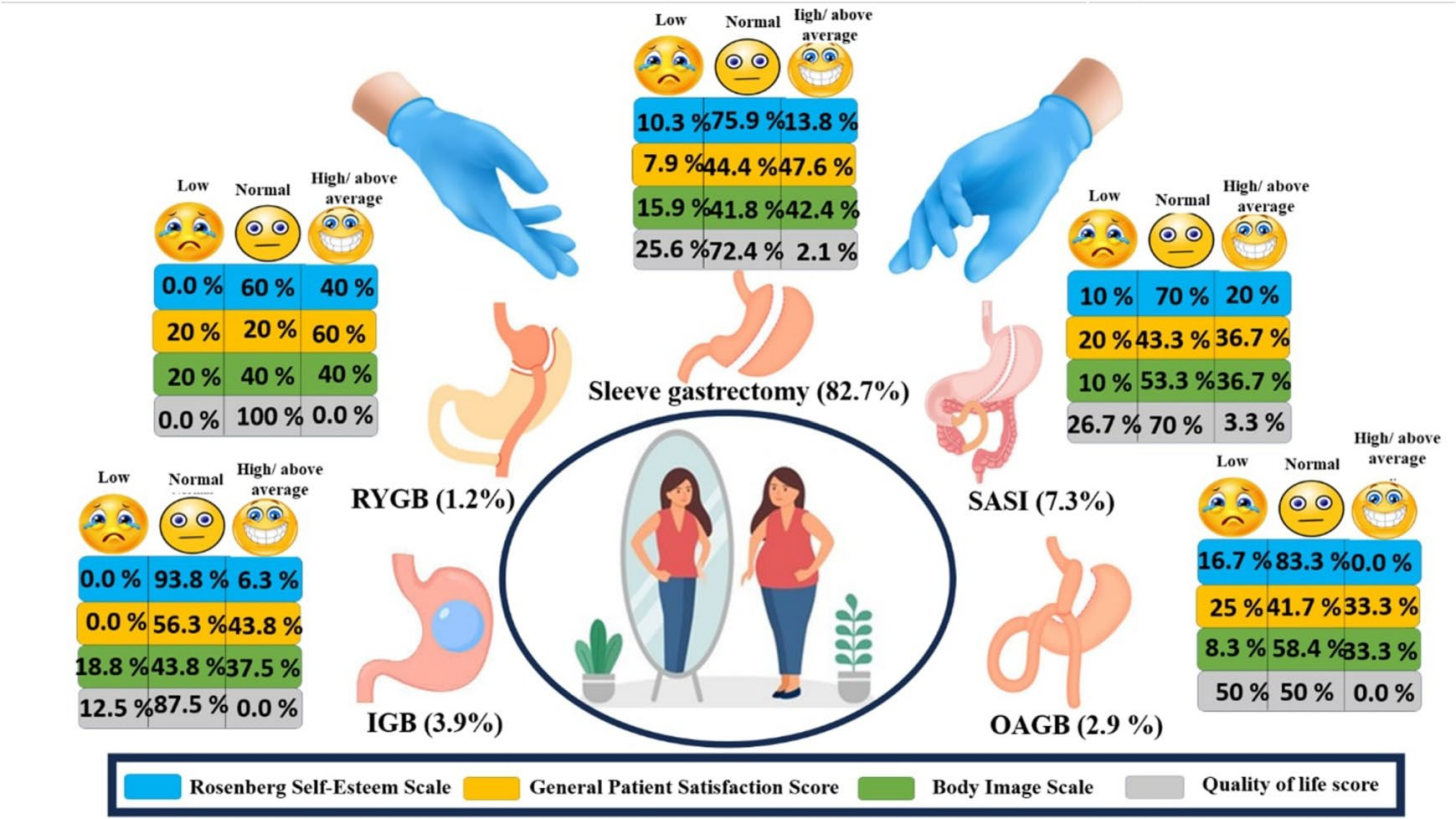
The types of bariatric interventions and the post-bariatric surgery (PBS) psychological assessment scores including the body image scale (BIS), General patient satisfaction score (GSABS), Rosenberg self-esteem (RSES), and quality of life score (QOLS)

The psycho-psychiatric assessment revealed significant differences in the Patient Satisfaction Score, Body Image Scale, and Rosenberg Self-Esteem Scale (*P* = 0.484). However, the quality-of-life score revealed a statistically significant difference *(P* = 0.049) in the median (IQR) between RYGB 114 (12–15) and OAGB 8.5 (8–12) when compared to other operations**.** Most SG patients, approximately 75%, had normal RSES and QOLS, while approximately 45% had high and above-average GSABS and BIS. IGB, SASI, and OAGB patients typically exhibit normal RSES, QOLS, GSABS, and BIS. However, the majority of RYGB patients had normal RSES (60%), QOLS (100.0%), BIS (40%), and high GSABS (60.0%) (Fig. 3).

## Discussion

This cross-sectional study examines the complexities of PBS psychosocial assessments across various BS backgrounds. Most participants experienced significant weight loss and improvement in their medical condition, with about one-fifth reporting self-limited complications. Participants generally showed normal scores on PBS QOLS, GSABS, and BIS, along with above-average RSES results.

Sleeve gastrectomy (SG) was the most common procedure, performed in 82.7% of cases, with less than 18% undergoing OAGB, IGB, and SASI. According to the IFSO worldwide survey from 2020–2021 [37], SG's popularity is due to its technical simplicity, shorter learning curve, lower nutritional deficiencies, fewer complications, and reduced cancer risk [38]. Our findings showed that SG patients had higher psychosocial assessment scores: 75% achieved normal RSES and QOLS, while around 45% had high GSABS and BIS scores compared to other surgeries. No significant differences were observed in total body weight loss percentage (TBWL%) or excess weight loss (EWL) across surgery types, indicating that all procedures were similarly effective in weight loss outcomes. This suggests that, at least within the scope of this study, all the surgeries were equally effective in achieving weight loss outcomes, as well as the effectiveness of a complete evaluation and choosing the right operation, regardless of when they were performed.

### Pre-operative trials of weight loss, and patient scores

Previous research has highlighted inconsistent links between RSES scores and pre-operative weight loss efforts. For instance, Barak et al. found that white patients, regardless of weight, had higher self-esteem while attempting to lose weight, whereas patients from darker ethnicities had higher self-esteem when not attempting to lose weight [39]. Conversely, Tolvanen et al. reported that individuals with multiple weight loss attempts and those who lost over 10 kg had higher RSES scores [40]. They also noted improved cognitive restraint in patients with repeated weight loss attempts, emphasizing the need for thorough assessments before bariatric surgery.

**Fig. 4:**
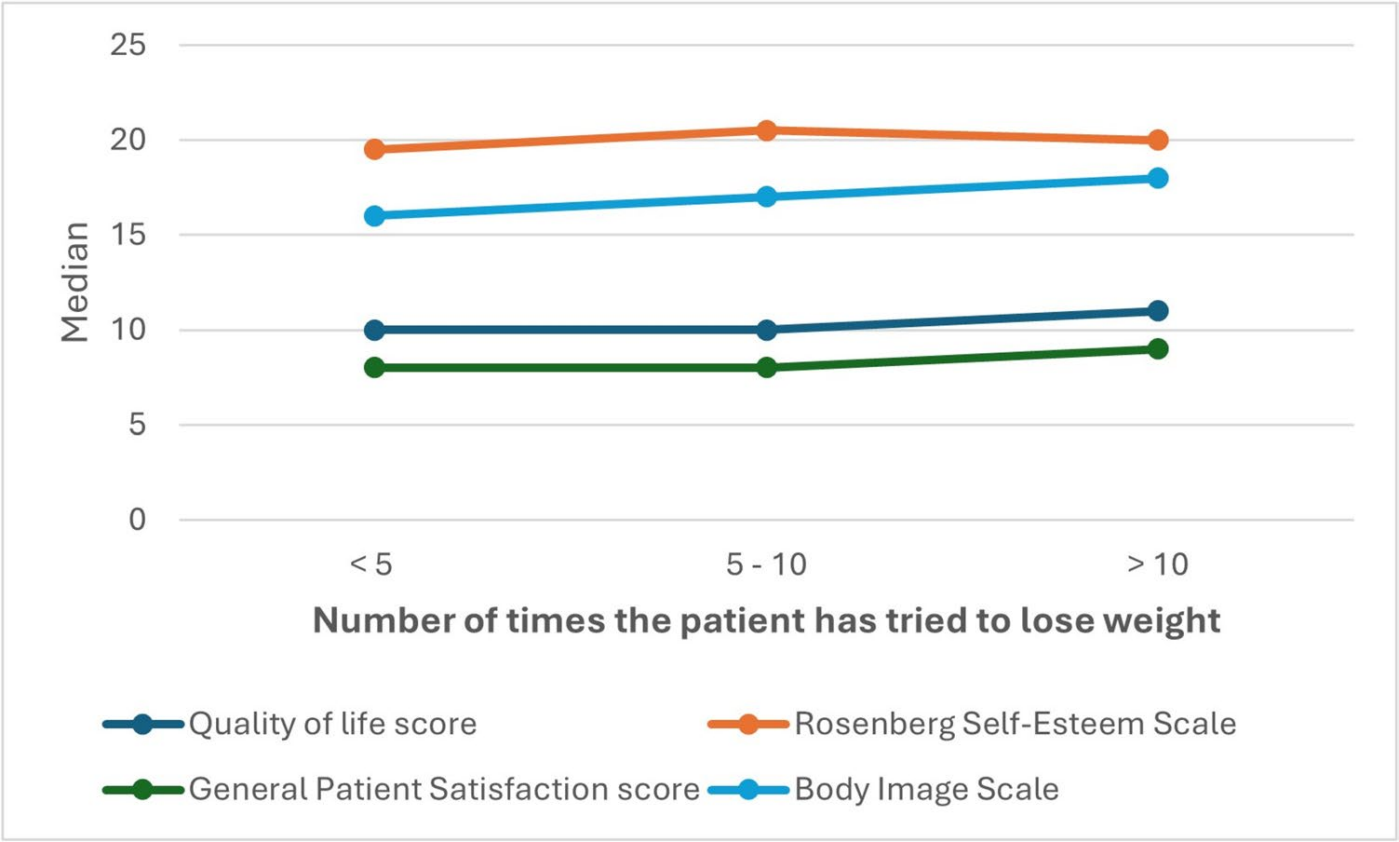
A line chart showing the correlation of the number of trials of weight loss to the median psychological assessment scores. Note that there is no correlation between the Rosenberg self-esteem (RSES), General patient satisfaction (GSABS), and quality of life scores (QOLS) with the number of trials of weight loss; however, there is a weak positive correlation between the number of trials and the body image scale (BIS)

**Fig. 5:**
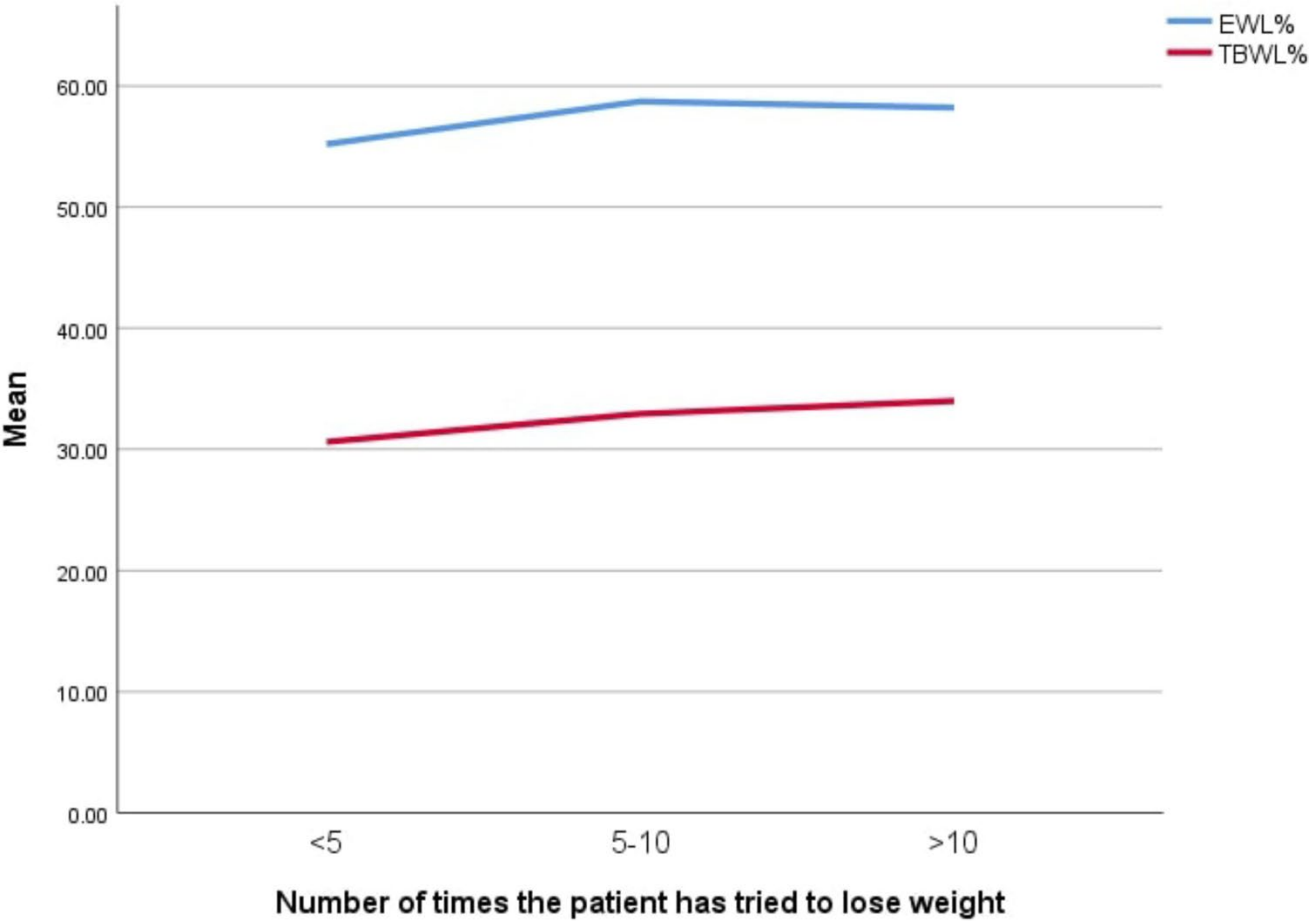
Line chart showing a correlation between EWL% (blue) (*p* = 0.148) and TBWL% (red) (*p* = 0.522) with the number of trials of weight loss

In our study, we found no significant correlation between RSES scores and the number of weight loss attempts (*p* = 0.460) (Table 5). While the number of attempts did not greatly affect patient satisfaction or RSES, it slightly influenced QOL scores (*p* = 0.058) and significantly impacted BIS scores (*p* = 0.008) (Fig. 4). Furthermore, our results showed no significance between the trials of weight loss and EWL% (*p* = 0.148) or TBWL% (*p* = 0.522) (Fig. 5). We attribute the noted significance between QOL, BIS, and the number of weight loss attempts to the sense of satisfaction experienced by patients after successfully losing weight after multiple attempts, which subjectively improved their QOL and body image perception.

Despite GSABS not being affected by the number of attempts, we speculate that gratitude for weight loss may enhance satisfaction, even with minor complications. Additionally, we hypothesize that RSES is more influenced by a patient’s baseline psychological condition rather than their weight loss attempts. Further studies using our methodology may clarify the relationship between baseline and post-operative RSES scores.

### The timing of intervention, weight loss, and patient scores

**Fig. 6:**
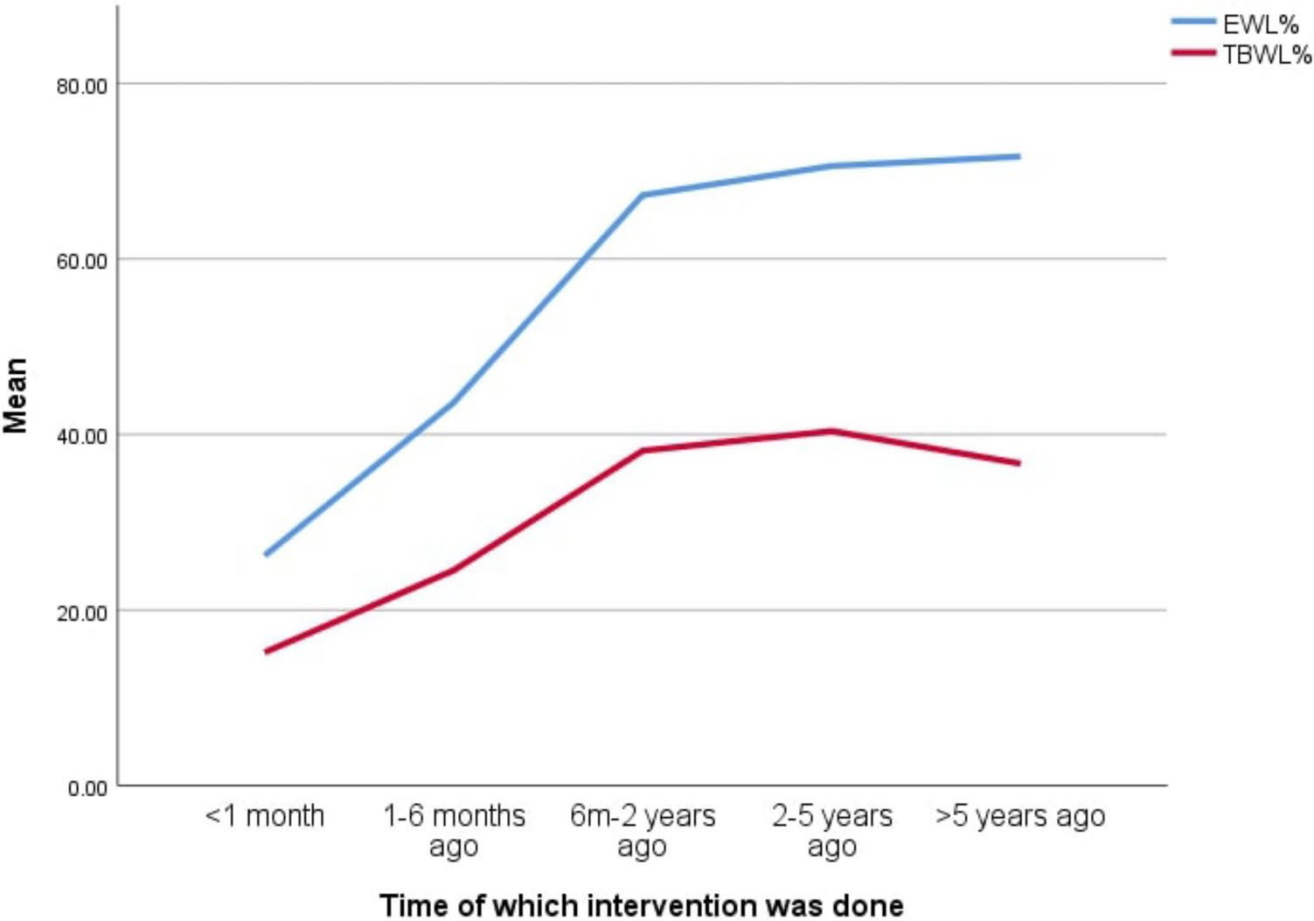
Line chart showing the trend of EWL% (blue) and TBWL% (red) concerning the timing of intervention

The time at which the interventions were done greatly impacted patients' scores (Fig. 1). The BIS scored higher on patients with more recent surgeries than on those with older surgeries, this may be because, in the first two years interval following BS, the patients tend to have higher EWL% and TBWL% than after this period [41] (Fig. 6). Furthermore, complications related to sagging of the skin, reflux, and recurrent weight gain [36], are more common after this period [42, 43].

On the contrary, our results indicate that more recent time intervals from surgery are associated with lower RSES scores, with a slight improvement of RSES scores as the time from surgery increases (Fig. 1). This could be explained by the discriminatory acts and ideologies targeted towards individuals with obesity. These obesity stigmata have been proven to affect patients' self-esteem and morbidity [44, 45]. However, as the body image improves, especially in the first two years, better RSES are achieved.

Although after the two-year interval, a steep drop in BIS scores was observed, we hypothesized that the continued improvement of RSES to the improved image of the patients in their clothes, rather than their skin sagging, As the previous obesity stigmata may have decreased slightly.

### PBS-related issues and PBS psycho-social assessment scores

Palumbo et al. observed a common trend in weight loss after bariatric surgeries, noting a rapid increase in TBWL% and EWL% in the first two years, followed by a slight decline [46]. This aligns with the drop in BIS scores over time and the observed EWL% and TBWL% (Table 5) (Supplementary File 2, Fig S2 and S3). Consequently, there was an improvement in QOLS and GSABS scores in the first two years, followed by stagnation and a slight decrease (Fig. 1).

Aldaqal et al. highlighted that 64 patients experienced noticeable skin sagging in the breast and abdomen within 12 months post-surgery, leading to dissatisfaction with body contours [43]. Hany et al. compared skin changes between patients who underwent bariatric surgery with ≥ 50% EWL% and those who had lost EWL% of ≥ 50% without index bariatric surgeries, finding significant reductions in skin elasticity and thickness in the surgery group, while collagen density remained unchanged [47]. These findings suggest that increased skin elasticity and decreased skin thickness attributed to weight loss could be the main concern for the decreased BIS noted after the two-year interval from BS in our study.

Although body contouring surgeries can enhance QOL [48], few patients pursue these options. Amarin et al. reported that while all surveyed patients with skin sagging post-surgery expressed interest, only 2.4% opted for body contouring [49], likely due to high surgical costs and concerns about post-operative complications.

Furthermore, our results showed no significant correlation with the intervention type, current age, or age at starting obesity. This finding suggests that the study's context does not fully capture or measure the complex nature of body image perception. Factors beyond PBS physical changes, such as emotional and psychological factors like personality, interpersonal factors, and social factors, could potentially affect the BIS.

In the Context of post bariatric surgery (PBS) RSES, our study noted that patients aged 30 to 45 reported a statistically higher PBS RSES. Additionally, those who rated high SSAH scores as excellent or very good, as well as those with an EWL% > 50%, reported a lower current weight and preoperative BMI. A variety of factors may be responsible for this, such as the associated improvement in the PBS QOLS physical and mental domains, higher GSABS scores, the PBS improvement of their health conditions (around 35% of post-BS patients reported complete weaning of their medications, an absolute recovery of their medical condition after a medical consultation), increased maturity, better-coping mechanisms, or a stronger sense of identity.

### The type of intervention and patient scores

We found no significance between the type of intervention and RSES, GSABS, and BIS. However, there was a significant correlation between the type of intervention and the QOL score. This correlation has been studied and adopted in different parts of the literature and for different interventions.

Several studies have investigated the impact of various bariatric surgeries on QOL, including IGB, SG, RYGB, and OAGB. However, there is limited data on the SASI operation due to its novelty. These studies have revealed discrepancies in the findings. A systematic review by Faria et al. found substantial QOL improvement following SG and RYGB [50], which agreed with an RCT that compared RYGB with SG, and showed no significance between the QOL of both groups [51]. In addition, Madani et al. have compared QOL scores between RYGB, SG, and OAGB, after five years from the interventions and found no significant results between these groups [52]. Fiorani et al. compared SG with RYGB over a one-year follow-up period and found better QOL results with SG than RYGB [53]. On the contrary, Mohos et al. found better QOL with RYGB than SG over a five year-follow-up period [54].

Rheinwalt et al. have similarly cross-sectionally compared QOL outcomes after both RYGB and OAGB, with a follow-up range of six to eleven years and found no significance between both groups [55].Other studies have linked the effects of weight loss while evaluating RYGB with the QOL, which observed a significant improvement of QOL when patients lose > 10% of their excess weight [56]. This had slight disparities with another study in Brazil that showed that QOL and weight loss improved in the short-term follow-up with a slight decrease of QOL and weight loss after a 7-year follow-up [57]. IGB did not fall from assessment, where a study measured the QOL with IGB concluded a significant improvement of QOL with IGB [58].

The effect of weight loss procedures on QOL has many variables such as reflux, weight loss, vomiting, dumping syndrome, and general health improvement. As more studies have favored RYGB in terms of QOL [54, 56, 57] Our data agrees with this statement, where RYGB scored significantly higher in terms of QOL than other surgeries.

These disparities in the results in the literature suggest that weight loss does improve the QOL regardless of the type of intervention that was undergone. However, further systematic reviews and meta-analyses are needed to provide evidence for such claims. Notably, our study did not provide a comprehensive comparison between the types of interventions and did not aim to provide this data. Rather our study provided the outcomes of bariatric procedures in general and has highlighted comparable observations that we have demonstrated for the literature.

### Strength and limitations

The study involved a diverse sample size with follow-up periods ranging from one month to over five years and included four types of bariatric surgeries. It utilized four internationally validated psychosocial assessment tools: BIS and RSES for self-esteem, SF-36 for quality of life, and GSABS for patient satisfaction and quality of life. While it provides key observations on weight loss and psychosocial health post-surgery to lay the groundwork for well-designed prospective studies, it has limitations typical of observational studies, such as recall bias and confounding factors. Notably, it is the first to use these four assessments together, aiming to generate hypotheses, but not their verification. Furthermore, this survey reflected the proportions of different BS occurring among bariatric centers, with the highest overall frequency of SG, which may impact the relationship between intervention types and patient scores. Although an online self-administered questionnaire helps limit bias, it may introduce self-report and sampling biases, affecting generalizability. Additionally, the absence of preoperative psychosocial assessments makes it challenging to differentiate between pre-existing conditions and those that arise as a result of surgery.

## Conclusion

PBS psycho-social assessments highlight the need to consider demographic and psychosocial factors in bariatric surgery outcomes. Patients aged 30 to 45 showed higher RSES, post-BS QOLS (SF-36), and GSABS. Those with a higher RSES experienced an EWL% of over 50%, underwent older interventions, and had lower current weights and pre-surgery BMIs. There was a weak negative correlation between current age, starting obesity, and post-BS QOLS. Males achieved higher scores on GSABS, TBWL%, and EWL%, with no significant relationship to intervention timing. The post-BS BIS showed no significant associations with weight loss or intervention type. No significant differences in TBWL% or EWL% were found among types of bariatric surgery, though most SG patients reported normal RSES and QOF, while IGB, SASI, MGB, and RYGB patients also displayed normal psychosocial measures.

We recommend psychologists, sociologists, and multidisciplinary teams should focus on the psychosocial effects of bariatric surgeries to enhance patient quality of life and outcomes. Both medical and psychosocial evaluations are essential before and after surgery. More research is needed to understand the factors influencing post-bariatric surgery outcomes and patient psychological well-being. Comparative studies of pre-and post-operative psychosocial scores with various interventions are necessary to identify the best options for patients. Additionally, the development and validation of a comprehensive psychosocial assessment tool and studies involving diverse samples from different bariatric surgery types, are recommended to assess their impacts accurately.

## Supplementary Information

Below is the link to the electronic supplementary material.

Supplementary file1 (DOCX 25 KB)

Supplementary file2 (DOCX 249 KB)

## Data Availability

All data generated in this article is available in the main article, Supplementary File 1 and Supplementary File 2.

## Abbreviations

BIS: Body Image scale
BS: Bariatric Surgery
GSABS: General Patient Satisfaction after Bariatric Surgeries Score
IGB: Intra-gastric Balloon
OAGB: One anastomosis Gastric Bypass
PBS: Post-Bariatric Surgery
QOLS: Quality of life score
RSES: Rosenberg self-esteem score
RYGB: Roux-en-Y Gastric Bypass
SASI: Single Anastomosis Sleeve Ileal Bipartition
SG: Sleeve Gastrectomy
SSAH: Subjective self-assessment, and health condition
EWL%: The Excess Body Weight Loss Percentage
TBWL%: The Total Body Weight Loss Percentage
