# Supplementary material for: The impact of weight loss after bariatric surgeries on the patient’s body image, quality of life, and self-esteem
Source: Langenbecks Arch Surg. 2025 Jan 4;410(1):24. doi: 10.1007/s00423-024-03568-6 (PMC11700042; doi:10.1007/s00423-024-03568-6)
Supplement: Supplementary file 1 — Supplementary file1 (DOCX 25 KB) [file 423_2024_3568_MOESM1_ESM.docx]

| Table S1 – Body Image Scale among participants post-bariatric Surgeries | | | | | |
| --- | --- | --- | --- | --- | --- |
| Body Image Scale | at a great deal  F(%) | Sometimes  F(%) | Rarely  F(%) | Never  F(%) | Total score |
|  |  |  |  |  |  |
| Are you self-conscious about your appearance | 268 (65.2) | 100 (24.3) | 29 (7.1) | 14 (3.4) | 2.51 ± 0.77 |
| Have you felt less physically attractive because of your weight? | 186 (45.3) | 115 (28) | 40 (9.7) | 70 (17) | 2.01 ± 1.11 |
| Were you dissatisfied with the way you look when you get dressed? | 136 (33.1) | 121 (29.4) | 37 (9) | 117 (28.5) | 1.67 ± 1.21 |
| Do you feel less feminine or masculine because of your weight? | 109 (26.5) | 96 (23.4) | 42 (10.2) | 164 (39.9) | 1.36 ± 1.25 |
| Do you find it difficult to look at yourself naked (without clothes)? | 130 (31.6) | 132 (32.1) | 36 (8.8) | 113 (27.5) | 1.68 ± 1.18 |
| Have you felt less sexually attractive because of your weight? | 146 (35.5) | 114 (27.7) | 48 (11.7) | 103 (25.1) | 1.74 ± 1.19 |
| Do you avoid people because of how you feel about your appearance? | 123 (29.9) | 100 (24.3) | 41 (10) | 147 (35.8) | 1.48 ± 1.25 |
| Do you feel that obesity has made your body less full? | 206 (50.1) | 102 (24.8) | 47 (11.4) | 56 (13.6) | 2.11 ± 1.07 |
| Have you ever felt dissatisfied with your body? | 187 (45.5) | 100 (24.3) | 47 (11.4) | 77 (18.7) | 1.96 ± 1.15 |
| Total score | 16.54 ± 6.27 | | | | |

Coding: at a great deal = 3 / Sometimes = 2 / Rarely = 1 / Never = 0

| Table S2 – Rosenberg Self-Esteem Scale among Participants post-bariatric Surgeries | | | | | |
| --- | --- | --- | --- | --- | --- |
| Rosenberg Self-Esteem Scale (RSES) | Strongly agree  F(%) | Agree  F(%) | Disagree  F(%) | Strongly disagree# F(%) | Total Score  mean ± SD |
| I feel like I am a valuable person, at least on an equal footing with others | 191 (46.5) | 193 (47) | 19 (4.6) | 8 (1.9) | 2.38 ± 0.67 |
| I feel that I have several good qualities | 252 (61.3) | 152 (37) | 4 (1) | 3 (0.7) | 2.59 ± 0.55 |
| In general, do you tend to feel that You are not beautiful?* | 22 (5.4) | 89 (21.7) | 204 (49.6) | 96 (23.4) | 1.91 ± 0.81 |
| Do you feel that you can do things as efficient as most of the people? | 203 (49.4) | 156 (38) | 34 (8.3) | 18 (4.4) | 2.32 ± 0.80 |
| I feel like you don't have much to be proud of.* | 43 (10.5) | 92 (22.4) | 164 (39.9) | 112 (27.3) | 1.84 ± 0.94 |
| Do you feel that you have a positive attitude towards yourself? | 207 (50.4) | 160 (38.9) | 32 (7.8) | 12 (2.9) | 2.37 ± 0.75 |
| In general, are you satisfied with yourself? | 176 (42.8) | 178 (43.3) | 43 (10.5) | 14 (3.4) | 2.25 ± 0.78 |
| Do You wish you had more respect for yourself?* | 185 (45) | 157 (38.2) | 35 (8.5) | 34 (8.3) | 0.80 ± 0.91 |
| Do you feel useless sometimes in general matters?* | 41 (10) | 81 (19.7) | 137 (33.3) | 152 (37) | 1.97 ± 0.98 |
| Do you sometimes think that you are not good at all?* | 57 (13.9) | 121 (29.4) | 134 (32.6) | 99 (24.1) | 1.67 ± 0.99 |
| Average Score for 411 patients | 20.11 ± 4.63 | | | | |

Coding: Strongly agree = 3 / Agree = 2 / Disagree = 1 / Strongly disagree = 0

Items with an asterisk are reverse-scored

| Table S3 – Quality of Life score, current health condition domain according to SF-36 among participants post-bariatric Surgeries. | | | | |
| --- | --- | --- | --- | --- |
| Current Health condition | There is no difficulty at all.  F(%) | I can but with little difficulty  F(%) | I can but with significant difficulty  F(%) | Total Score  mean ± SD |
| Can you do moderate-strength activities (moving a table - or carrying a broom)? | 266 (64.7) | 125 (30.4) | 20 (4.9) | 1.59 ± 0.58 |
| Can you climb three or four floors on the stairs? | 224 (54.5) | 143 (34.8) | 44 (10.7) | 1.44 ± 0.68 |
| Total Score | 3.04 ± 1.09 | | | |

Coding: There is no difficulty at all = 2 / I can, but with little difficulty = 1 / I can but with a significant difficulty = 0

| Table S4 – Quality of Life score mental and emotional well-being domain according to SF-36 among participants post-bariatric Surgeries. | | | | | |
| --- | --- | --- | --- | --- | --- |
| Current mental and emotional well-being | All the time  F(%) | Most of the time  F(%) | Sometimes  F(%) | Rarely  F(%) | Total Score  mean ± SD |
| Do you experience Any pain that interferes with your normal work (inside and outside the home)? | 16 (3.9) | 61 (14.8) | 92 (22.4) | 242 (58.9) | 0.64 ± 0.87 |
| Do you feel calm and peaceful? | 110 (26.8) | 156 (38) | 92 (22.4) | 53 (12.9) | 1.78 ± 0.98 |
| Do you experience any depression or sadness? | 22 (5.4) | 86 (20.9) | 82 (20) | 221 (53.8) | 0.78 ± 0.96 |
| Do you suffer from physical, psychological, or emotional health problems? | 68 (16.5) | 128 (31.1) | 109 (26.5) | 106 (25.8) | 1.38 ± 1.04 |
| Do you think you are achieving less than you would like? | 39 (9.5) | 111 (27) | 111 (27) | 150 (36.5) | 1.09 ± 1 |
| Do you believe that you are performing your activities and work efficiently as usual? | 108 (26.3) | 158 (38.4) | 73 (17.8) | 72 (17.5) | 1.73 ± 1.03 |
| Total Score  mean ± SD | 7.42 ± 2.87 | | | | |

Coding: All the time = 3 / Most of the time = 2 / Sometimes = 1 / Rarely = 0

| Table S5 – Post Bariatric Surgery Score according to SF-36 among participants post-bariatric Surgeries | | | | | | | |
| --- | --- | --- | --- | --- | --- | --- | --- |
| After the operation and weight loss, | **Has significantly increased** | **Has increased** | **Same effort (has not been affected)** | **Has been slightly affected (lessened)** | **Has Lessened significantly** | **I prefer not to answer** | Total Score  mean ± SD |
| I feel that my physical activity has: | 49 (11.9) | 233 (56.7) | 49 (11.9) | 55 (13.4) | 25 (6.1) | - | 2.55 ± 1.06 |
| I feel that my social activity and social inter-activeness have: | 52 (12.7) | 230 (56) | 75 (18.2) | 34 (8.3) | 20 (4.9) | - | 2.63 ± 0.97 |
| I feel that my ability to work has: | 43 (10.5) | 214 (52.1) | 78 (19) | 58 (14.1) | 18 (4.4) | - | 2.50 ± 1 |
| , I feel that my sexual ability/ libido has: | 28 (6.8) | 160 (38.9) | 135 (32.8) | 21 (5.1) | 17 (4.1) | 50 (12.2) | 2.45 ± 0.89 |
| Total Score  mean ± SD | 9.83 ± 3.40 | | | | | | |

Coding: Has significantly increased = 4 / Has increased = 3 / Same effort (has not been affected) = 2 / Has been slightly affected (lessened) = 1 / Has Lessened significantly = 0

Table S6: General Patient Satisfaction Score After Bariatric Surgeries (GSABS) among participants post-bariatric Surgeries

| **General Patient Satisfaction Score After Bariatric Surgeries (GSABS)** | **Very satisfied** | **Satisfied** | **Borderline/neutral** | **Not Satisfied** | Total Score  mean ± SD |
| --- | --- | --- | --- | --- | --- |
| Regarding the overall operation experience in terms of the preparation for surgery, post-operative pain, post-operative hospital stays, and outpatient clinic follow-up, you are: | 275 (66.9) | 101 (24.6) | 31 (7.5) | 4 (0.9) | 2.58 ± 0.66 |
| Regarding The shape of your body after the operation, you are: | 188 (45.7) | 146 (35.5) | 67 (16.3) | 10 (2.4) | 2.24 ± 0.81 |
| Regarding The appearance of your skin after the operation, you are: | 58 (14.1) | 100 (24.3) | 163 (39.7) | 90 (21.9) | 1.31 ± 0.97 |
| Regarding your total weight loss, you are: | 160 (38.9) | 110 (26.8) | 103 (25.1) | 38 (9.2) | 1.95 ± 1.01 |
| Total Score  mean ± SD | 8.08 ± 2.39 | | | | |

Coding: Very satisfied = 3 / Satisfied = 2 / Somehow satisfied = 1 / Not Satisfied = 0
