# Supplementary material for: The impact of weight loss after bariatric surgeries on the patient’s body image, quality of life, and self-esteem
Source: Langenbecks Arch Surg. 2025 Jan 4;410(1):24. doi: 10.1007/s00423-024-03568-6 (PMC11700042; doi:10.1007/s00423-024-03568-6)
Supplement: Supplementary file 2 — Supplementary file2 (DOCX 249 KB) [file 423_2024_3568_MOESM2_ESM.docx]

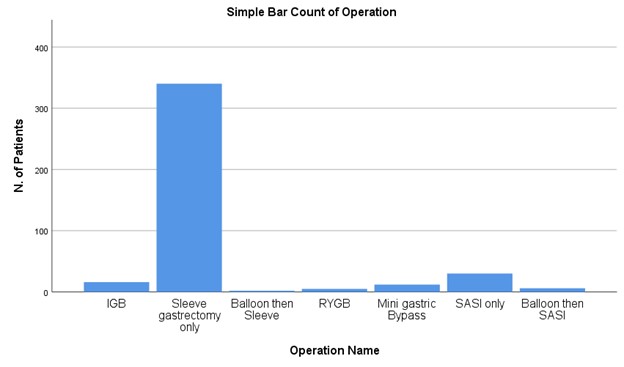


**Figure S1: The frequency of bariatric surgery among participants from a single obesity control setting**


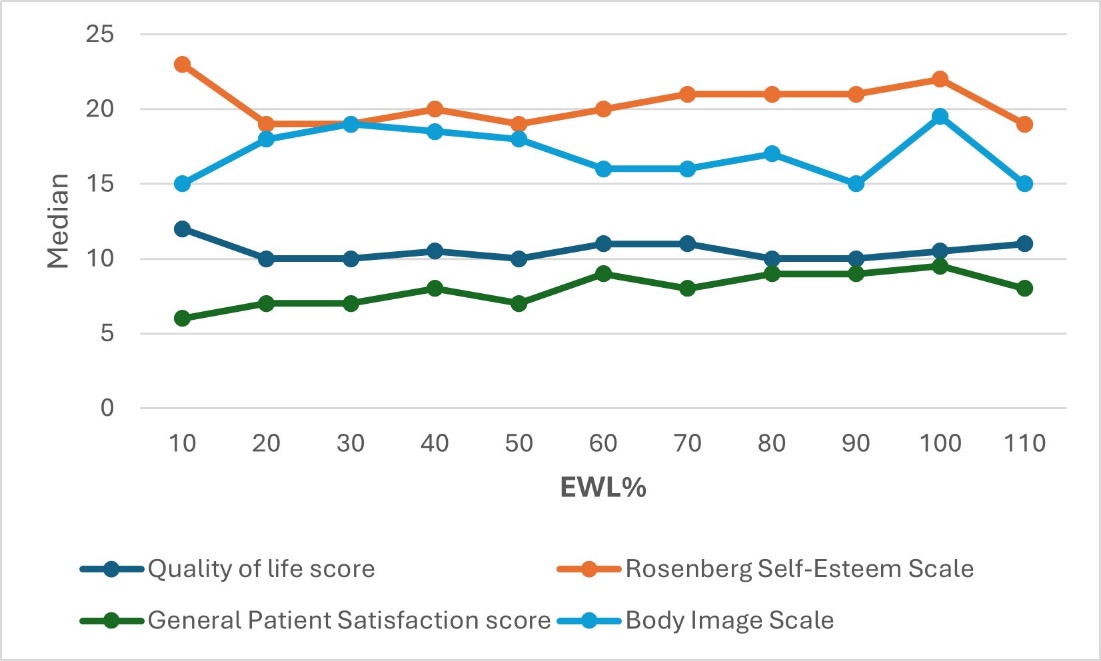


**Figure S2: A line chart showing no correlation between the Excess Weight loss Percentage (EWL%) and the median psychological assessment scores.**


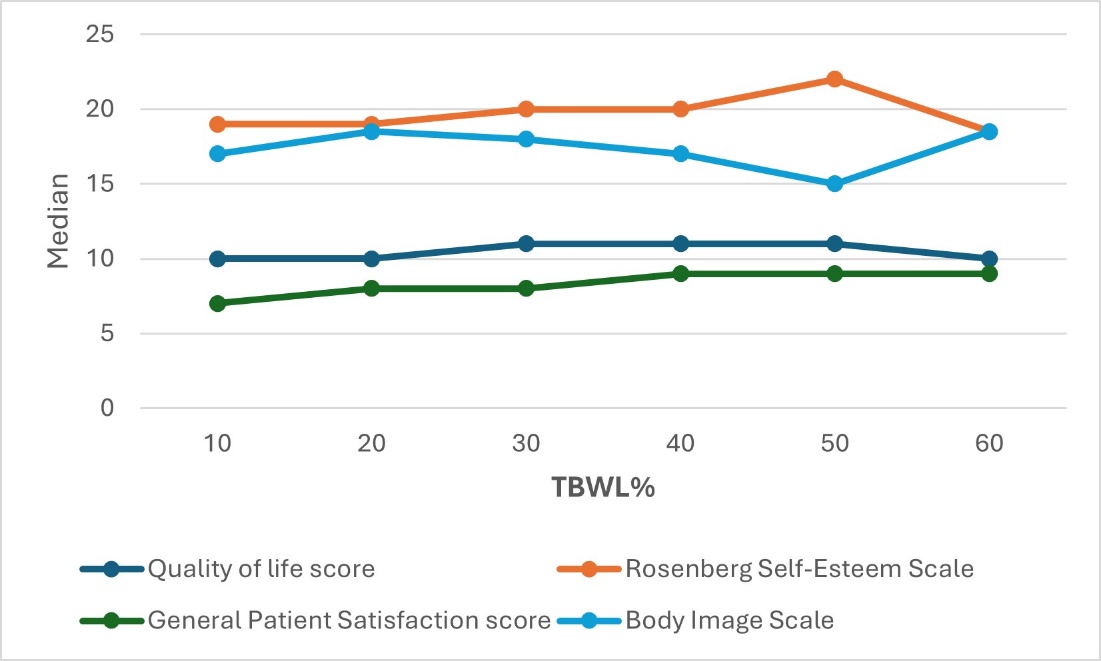


**Figure S3: A line chart showing no correlation between the Total Body Weight loss Percentage (TBWL%) and the median psychological assessment scores.**
